# Supplementary material for: Distinct shared and compartment-enriched oncogenic networks drive primary versus metastatic breast cancer
Source: Nat Commun. 2023 Jul 18;14:4313. doi: 10.1038/s41467-023-39935-y (PMC10354065; doi:10.1038/s41467-023-39935-y)

Distinct shared and compartment-enriched oncogenic networks drive primary versus metastatic breast cancer

Zhe Jiang[1], YoungJun Ju[1,*], Amjad Ali[1,*], Philip E.D. Chung[1,2,*], Patryk Skowron[2,3,4,*], Dong-Yu Wang[1,*], Mariusz Shrestha[1], Huiqin Li[1], Jeff C. Liu[5], Ioulia Vorobieva[1], Ronak Ghanbari-Azarnier[1,2], Ethel Mwewa[1], Marianne Koritzinsky[6], Yaacov Ben-David[7,8], James R. Woodgett[9], Charles M. Perou[10], Adam Dupuy[11], Gary D. Bader[5,12], Sean E. Egan[3,12], Michael D. Taylor[2,3,4] and Eldad Zacksenhaus[1,2,13,**]

Supplementary Figures S1-11, legends & uncropped Western blots

a

SB primary gCISs	SB mets gCISs
Met	Met
Exoc6b	Prlr
Plag1	**Wdr33**
Prlr	Srgap2
Nf1	Sncaip
Jup	Nf1
Foxp1	Lrch3
Nfib	**Fbxw4**
Kdm6a	Add3
Fbxw7	Tnrc6b
Wwc1	Stat5b
Ptpn4	Pten
Nipbl	Pbrm1
Fbxw11	Jup
Rasa1	Hpd
Pparg	Grhl1
Mllt10	Cyp2c67
En2	**Cdc42bpa**
Dmxl1	Ubxn7
Chd1	Stag2
Chchd7	Ppfibp1
Top1	Ncoa6
Tgfbr1	Mtmr3
St5	Lrrc56
Spnb2	Hras1
Smad4	Ep300
Slain2	Asxl2
Sfi1	Abcb7
Prr5l	Zfml
Polk	Vps54
Pdzd8	Tm9sf3
Pds5a	Stau1
Mll5	Stat5a
Map3k3	Spop
Larp1	Slc24a4
Itch	Sfi1
Fat1	Sec24a
Erp44	Rft1
Dear1	Pspc1
Clock	Prkacb
Zfx	Plk4
Vkorc1l1	Nsun5
Ugcg	Notch1
Ube2n	Mid2
Tsix	Map3k3
Trit1	Lrig2
Stx6	Hectd1
Stat5b	Ep400
Socs5	Eaf2
Slc35d1	Dlgap4
Setx	Csnk1g3
Sema4d	Crem
Rlf	Btbd1
Rbl1	Atf2
Ppp2r1a	Arf3
Pdcd4	Ankhd1
Pafah1b1	Zzz3
Nup153	Zranb1
Notch1	Zfp677
Madd	Wasf2
Lats1	Ube2d3
Itgb3	Rprd1b
Hnrnpm	Rab18
Ggnbp2	Prpf6
Fbxo34	Osbpl11
Fam117a	Olfr1457
Evi2b	Naa15
Erbb2	Ints2
Cyth1	Grb2
Clic4	Golgb1
Cept1	Gm6225
Cdc40	G3bp2
Capza2	Fnbp4
Azin1	Ets1
Ahnak	Cyld
	Cwc22
	Chia
	Cep110
	Cdc5l
	2700078E11Rik

b

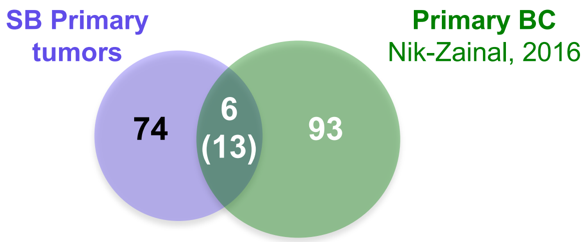

Nf1, FoxP1, Kdm6a, Fbxw7, Smad4, Notch1
Rb – RB1, CCND1, CCNE1, CDKN2A, CDKN2B, CDK6, CDKN1B

c

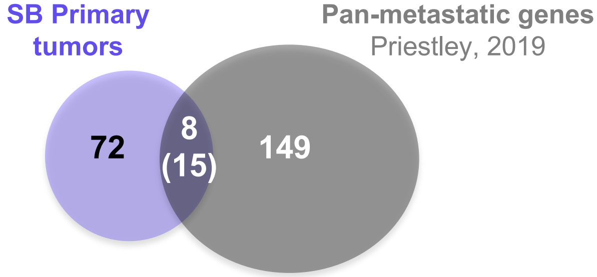

Met, Nf1, Foxp1, Kdm6a, Chd1, Smad4, Fat1, Erbb2
Rb – RB1, CCND1, CCND2, CCNE1, CDKN1B, CDKN2A, CDKN2C

d

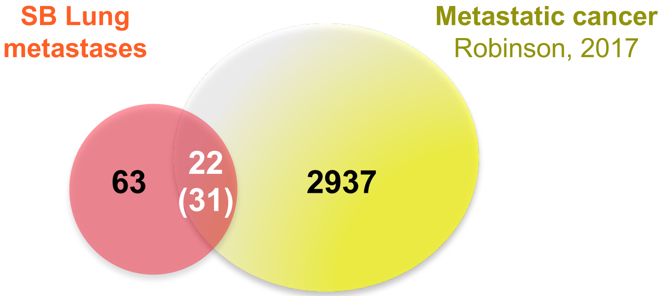

Sncaip, Nf1, Tnrc6b, Stat5b, Pten, Pbrm1, Ep300, Asxl2, Tm9sf3, Sec24a, Rft1, Pspc1, Prkacb, Notch1, Map3k3, Dlgap4, Atf2, Arf3, Ankhd1, Zzz3, Zranb1, Naa15
Rb – RB1, CCND1, CCND2, CCND3, CCNE1, CDKN2A,CDKN2C, CDK4, CDK6

e

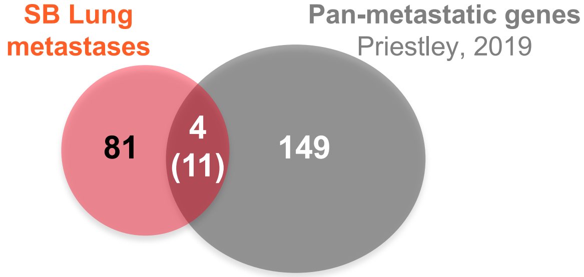

Met, Nf1, Pten, Asxl2
Rb – RB1, CCND1, CCND2, CCNE1, CDKN1B, CDKN2A, CDKN2C

Supplementary Fig. S1

Supplementary Figure S1 Related to Figure 1. **a.** List of primary and metastasis gCISs. S-drivers are highlighted in red, and the highly frequent, representative gCISs from the three hubs (WDR33, FBXW4, and CDC42BPA) are in blue (arrows). **b-e.** Venn diagram showing the overlap between gCISs identified in our SB screens and oncogenic alterations found in primary (b, c) and metastatic (d, e) breast cancer patients in indicated studies.

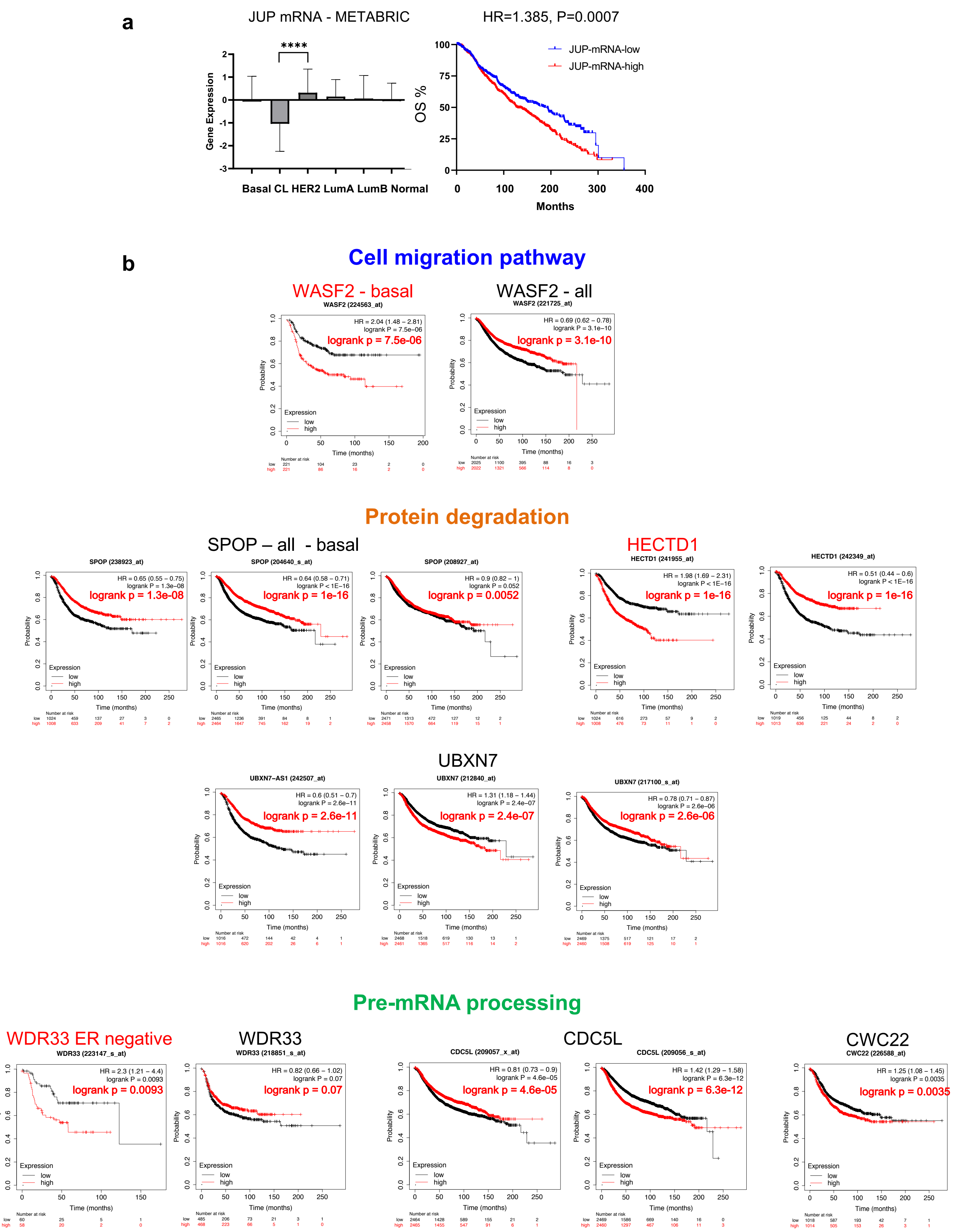

Supplementary Fig. S2a-b

c

Metastasis-specific drivers

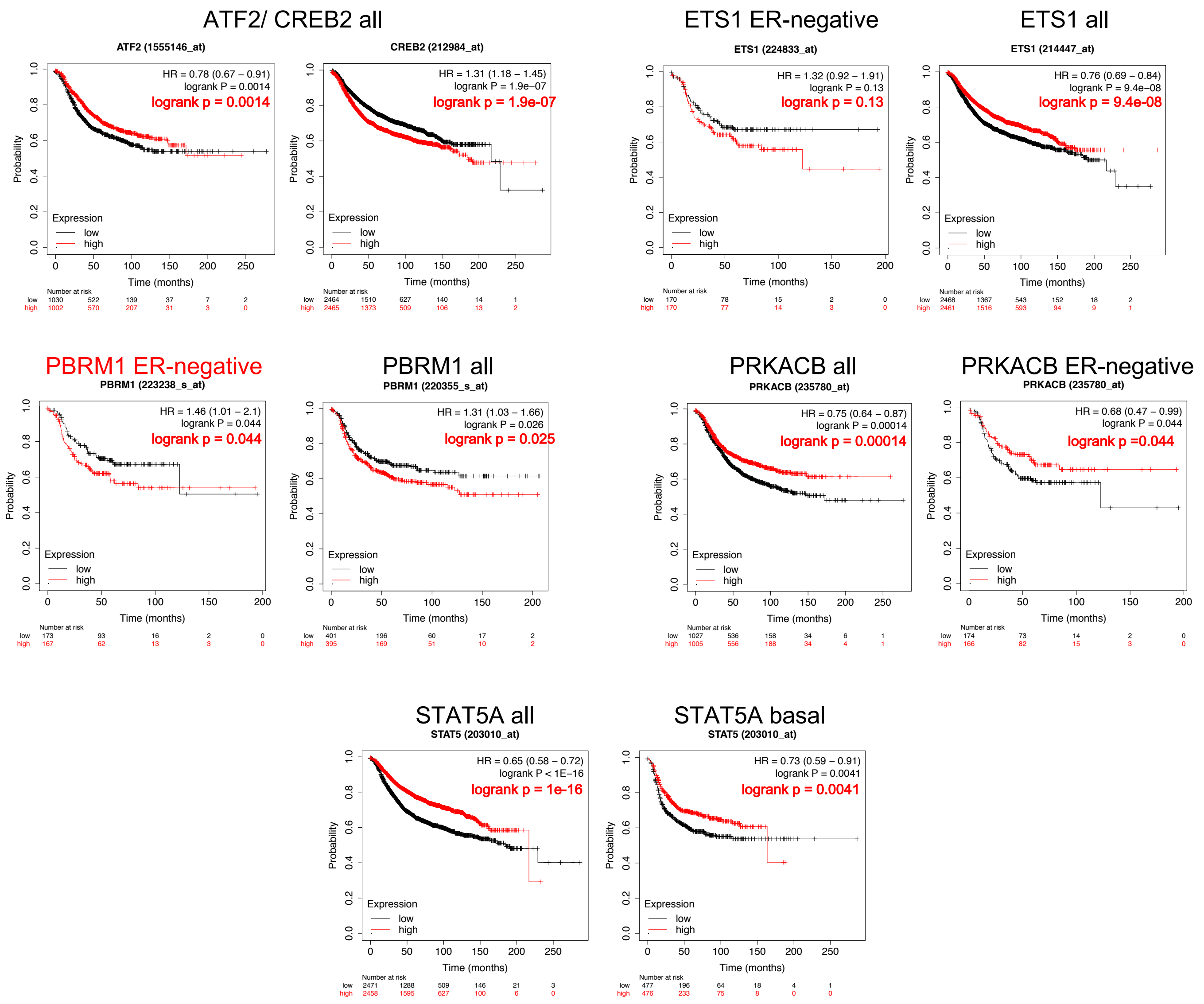

Supplementary Figure S2 Related to Figure 3. **a.** Expression of JUP in different breast cancer subtypes, showing lowest expression in claudin-low (left), and Kaplan-Meier overall survival (OS) of breast cancer patients segregated based on JUP mRNA level (right). Error bars represent SD.

b. relapse-free survival (RFS) based on gCISs from the cell migration pathway, protein degradation and pre-mRNA processing hubs (Fig. 2e). **c.** RFS based on gCISs from the metastasis-specific migration pathway (Fig. 3b). Analysis of other genes is shown in Figs. 6g and S5. Note that different probes capturing different splice forms exhibit distinct RFS curves. Highlighted in red are RFS curves shown in Figure 3c.

Supplementary Fig. S2c

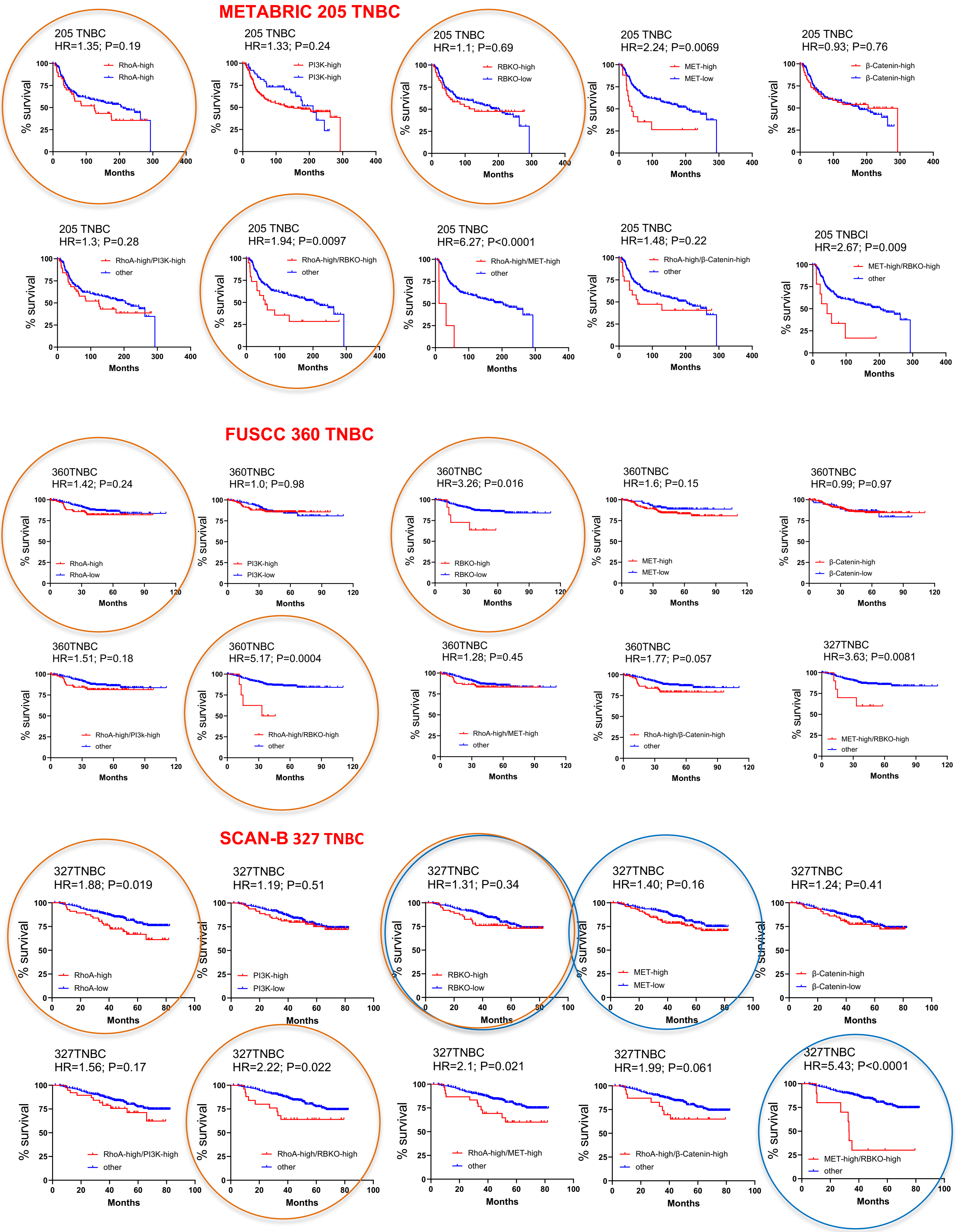

Supplementary Fig. S3

Supplementary Figure S3 Related to Figures 4h and 8a. Kaplan-Meier overall survival curves of TNBC patients segregated based on RhoA, PI3K RBKO-high (loss), MET or b-catenin pathway activity (top) or combination thereof (bottom) in three independent clinical databases: METABRIC, FUSCC and SCAN-B. Curves shown in Figure 4h and 8a are circled.

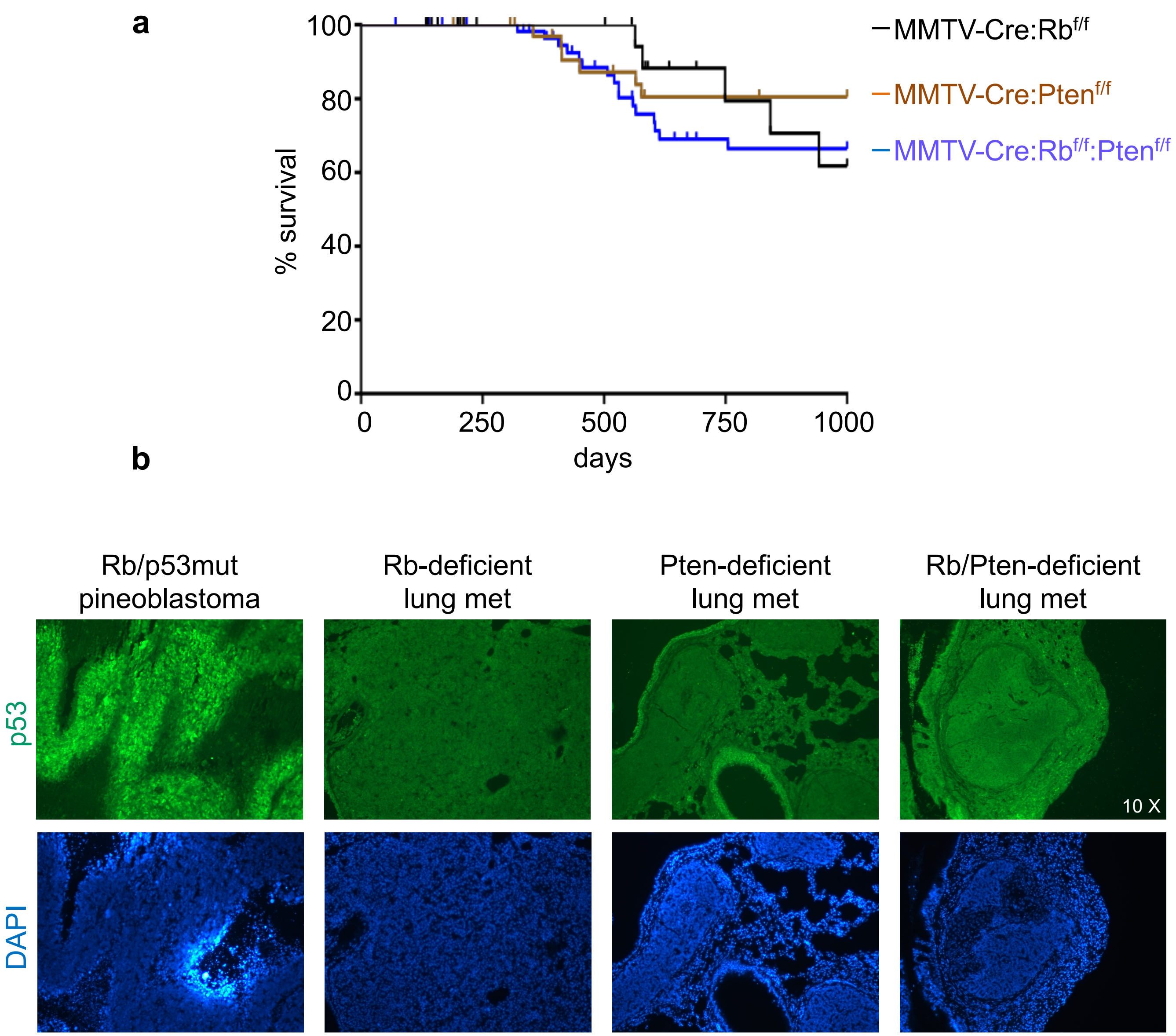

Supplementary Figure S4 Related to Figure 5b. a. Kinetics of primary mammary tumor growth in MMTV-Cre:$Rb^{f/f}$, MMTV-Cre:$Pten^{f/f}$ and MMTV-Cre:$Rb^{f/f}$:$Pten^{f/f}$ mice, and **b.** expression of p53 in highly metastatic lung sections from MMTV-Cre:$Rb^{f/f}$:$Pten^{f/f}$ mice. Sections through mouse pineoblastoma driven by Rb-loss plus p53-R270H mutation that stabilizes this dominant-negative tumor suppressor oncogenic allele was used as a positive control[117].

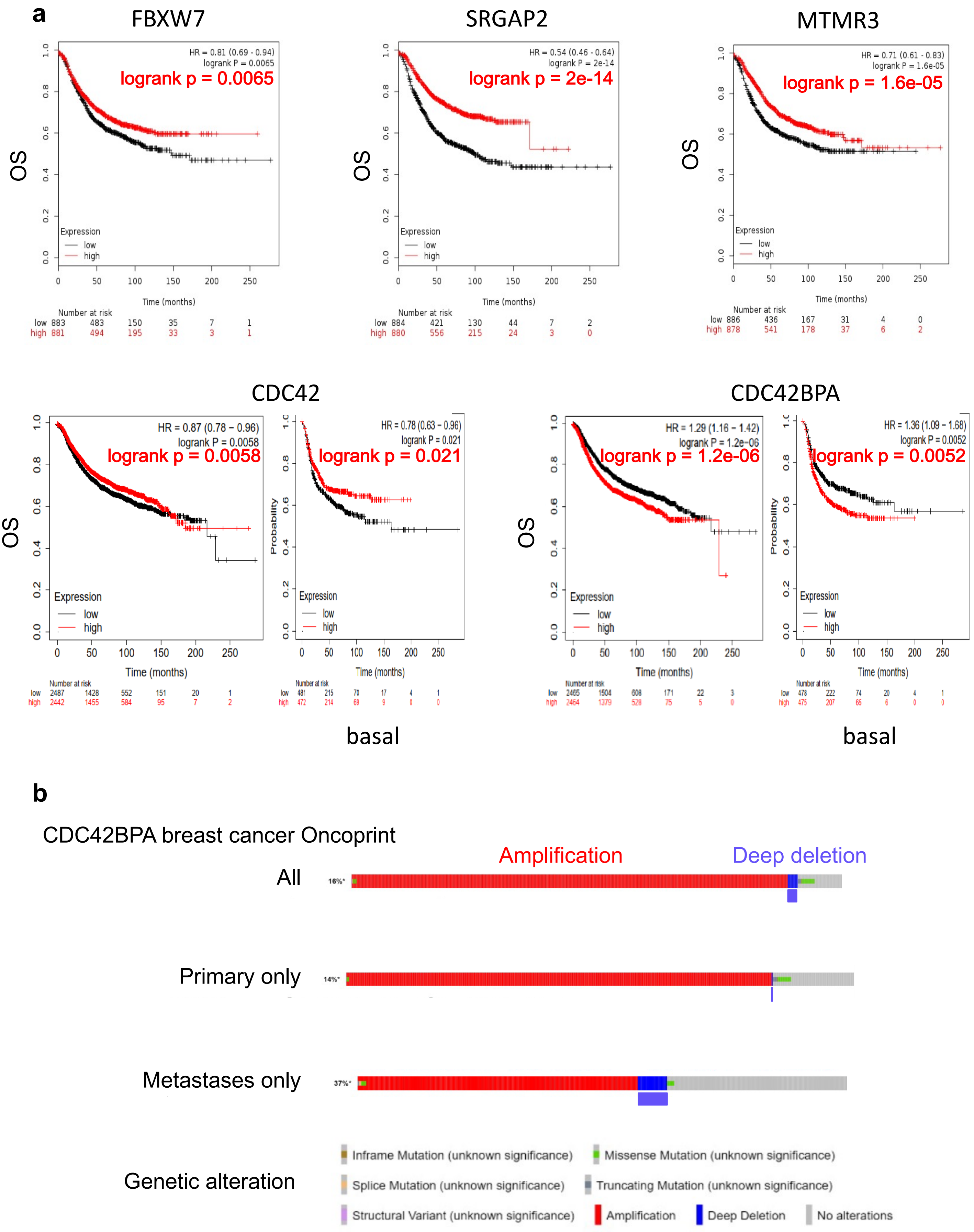

Supplementary Fig. S5

Supplementary Figure S5 Related to Figure 5e. a. Kaplan-Meier overall survival (OS) curves for breast cancer patients stratified on the basis of *FBXW7, SRGAP2, MTMR3*, *CDC42 or CDC42BPA* mRNA gene expression. For *CDC42* and *CDC42BPA,* OS curves are also shown for basal-like breast cancer patients as indicated. B. Oncoprint analysis of CDC42BPA showing amplification (red) or Deep deletion (blue) in all samples, primary-only breast cancer or metastatic only lesions. Note elevated deletion rate in metastatic samples.

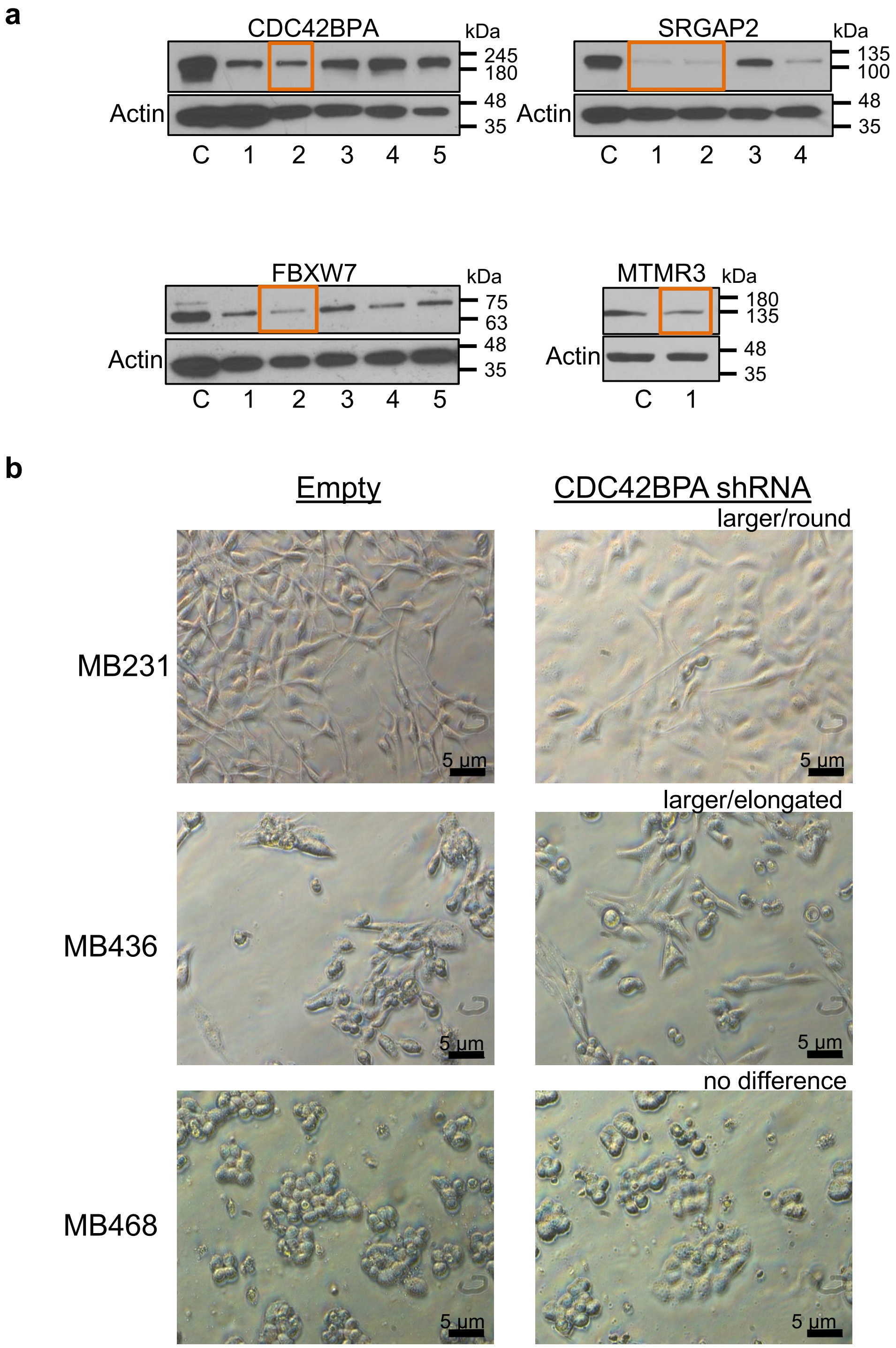

Supplementary Figure S6 Related to Figure 5e, g. **a.** Identification of lenti-shRNA clones that efficiently knockdown CDC42BPA, SRGAP2, FBXW7 or MTMR3 in MDA-MB-436 TNBC cells. Red boxes demarcate most effective lenti-shRNA and derived cell lines used in this study. **b.** Images of indicated TNBC cell lines transduced with CDC42BPA lenti-shRNA or empty control virus. CDC42BPA-depleted MDA-MB-231 and MDA-MB-436 cells are larger and rounder or more elongated, respectively, whereas MDA-MB-468 are similar to control cells.

Supplementary Fig. S6

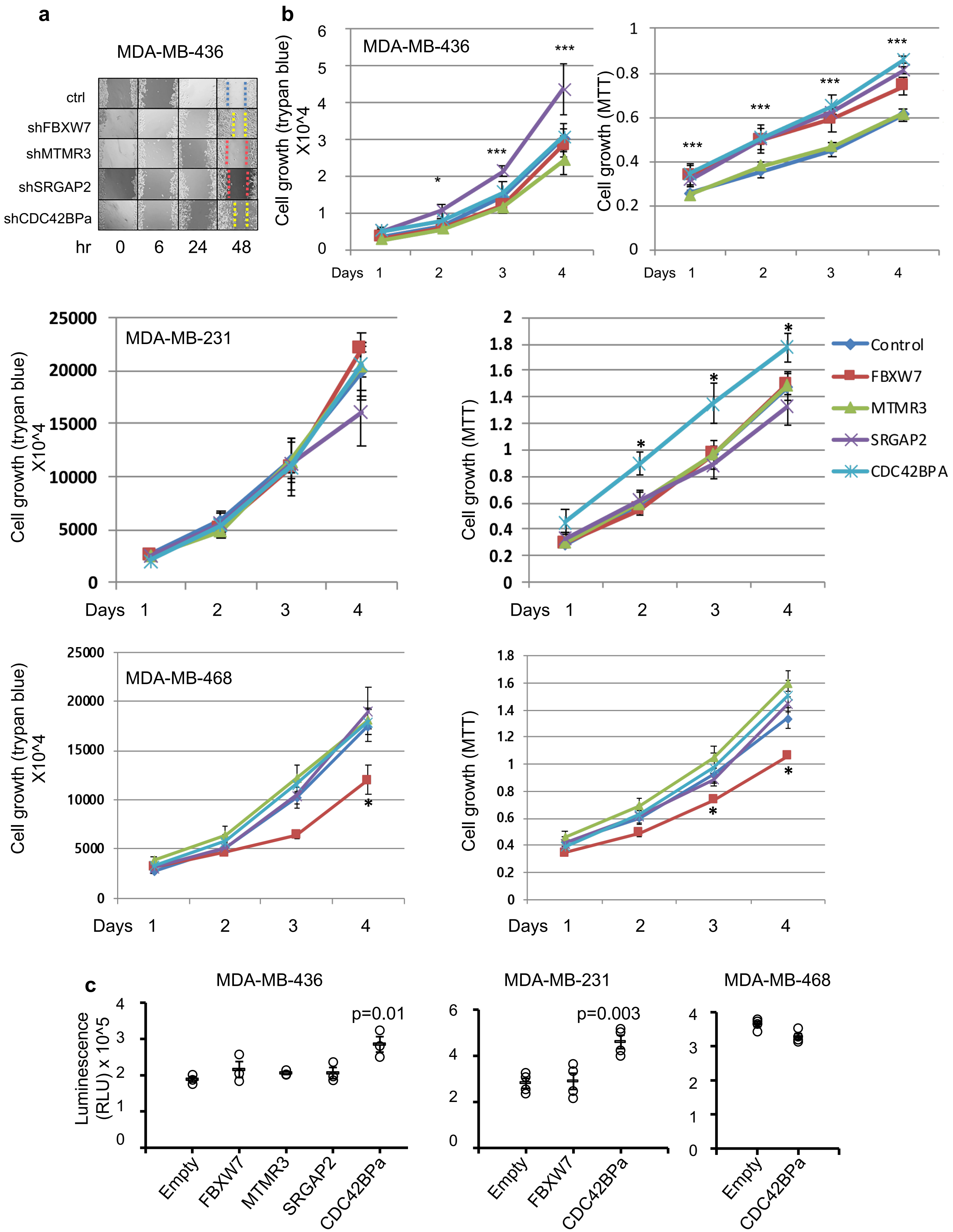

Supplementary Fig. S7a-c

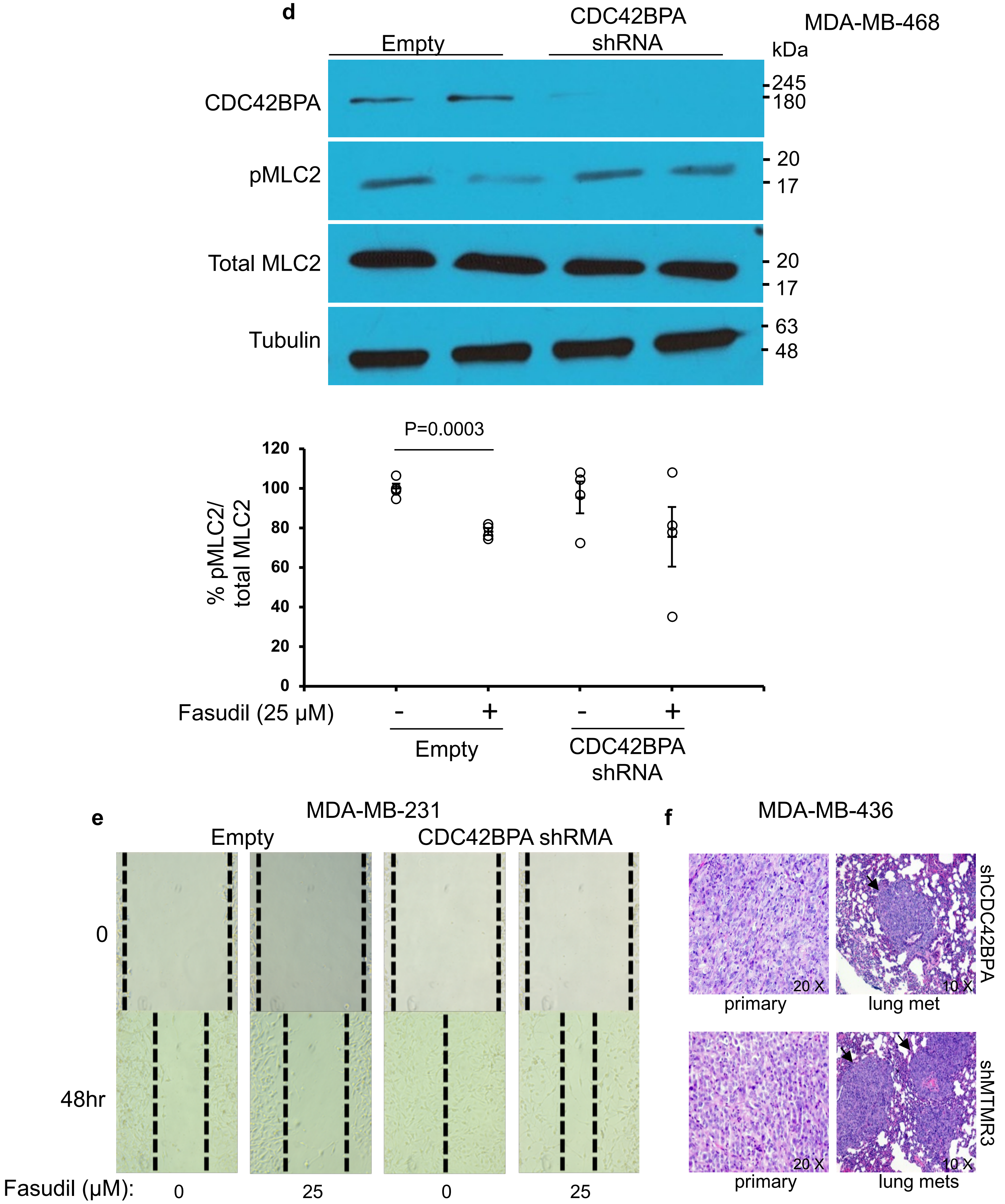

Supplementary Fig. S7d-f

Supplementary Figure S7 Related to Figure 5e, f, h. a-c. Representative effects of *FBXW7, SRGAP2, CDC42BPA* or *MTMR3* knock-down on TNBC cell migration (a), survival by trypan blue exclusion analysis vs cell growth/proliferation by MTT assays (b), and total ATP levels (c) in indicated TNBC cell lines in 3-4 technical replicates each of 3-4 biological replicates. P values determined by unpaired, two-tailed student t tests. **d.** Top, MDA-MB-468 cells stably transduced with empty or CDC42BPA lenti-shRNA virus and treated with Fasudil (25uM) or vehicle alone followed by western blots for CDC42BPA, anti-pMLC2-Thr18/Ser19 or total MLC2 with Tubulin serving as loading control. Bottom, statistical analysis on four independent biological replicates; P value calculated by two-tailed student t-test. **e.** A representative scratch assay of MDA-MB-231 (Fig. 5f for statistical analysis). **f.** Representative H&E staining of primary tumors and lung mets following transplantation of MDA-MB-436 cells depleted for *CDC42BPA* or *MTMR3* into NSG mice. Error bars in panels b-d represent SD.

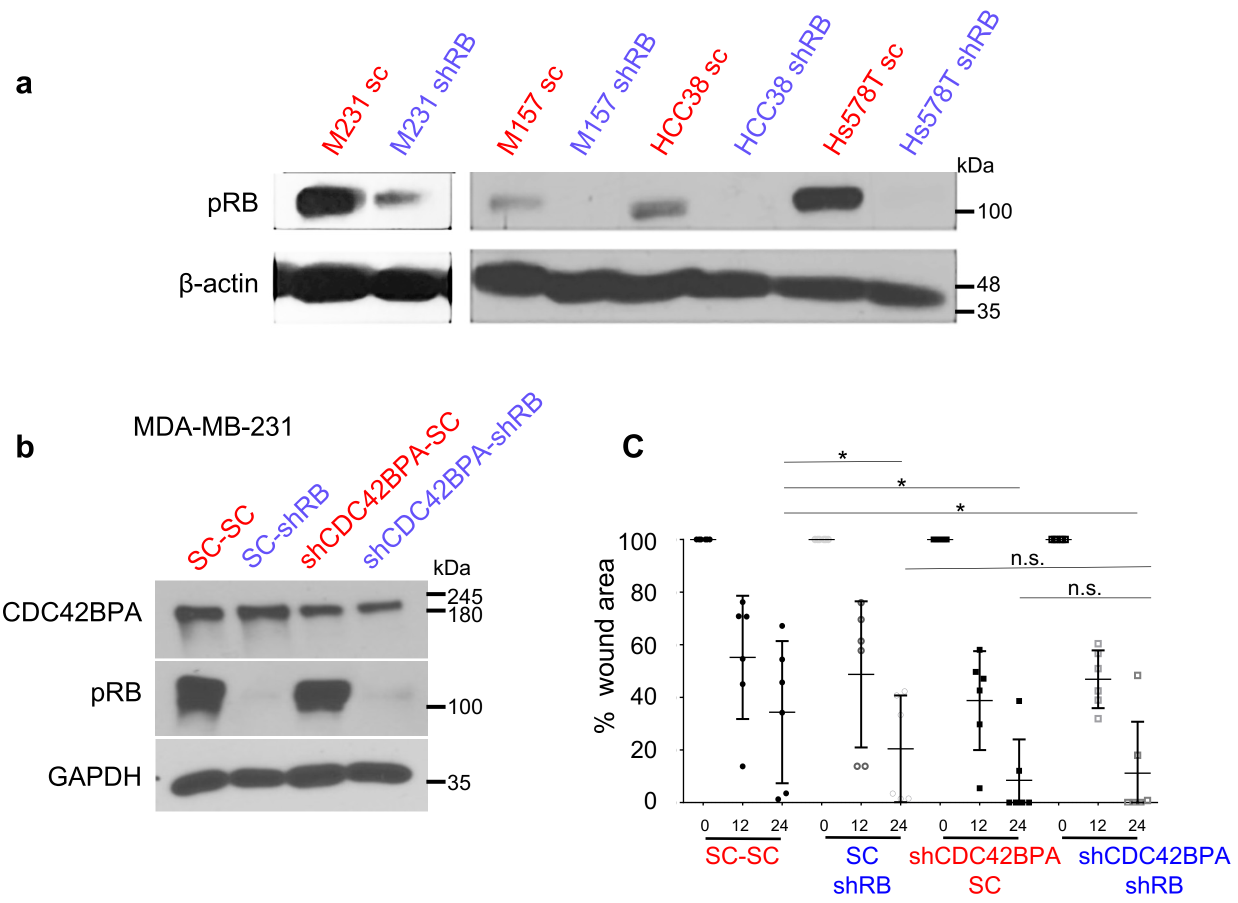

Supplementary Figure S8 Related to Figure 5f. **a.** Western blot analysis showing the efficacy of RB knockdown in indicated cell lines. **b.** Western blot analysis showing the generation of isogenic MDA-MB-231 cell lines with depletion of RB, CDC42BPA or both proteins. GAPDH was used as loading control. **c.** Migration analysis by scratch-wound assays of isogenic MDA-MB-231 cell lines depleted for RB, CDC42BPA or both proteins versus scrambled control cells. * denotes $P<0.05$ by two-tailed student t-test ($n=6$) from a single experiment, representing three biological replicates. n.s. not significant. Error bars represent SD.

Supplementary Fig. S8

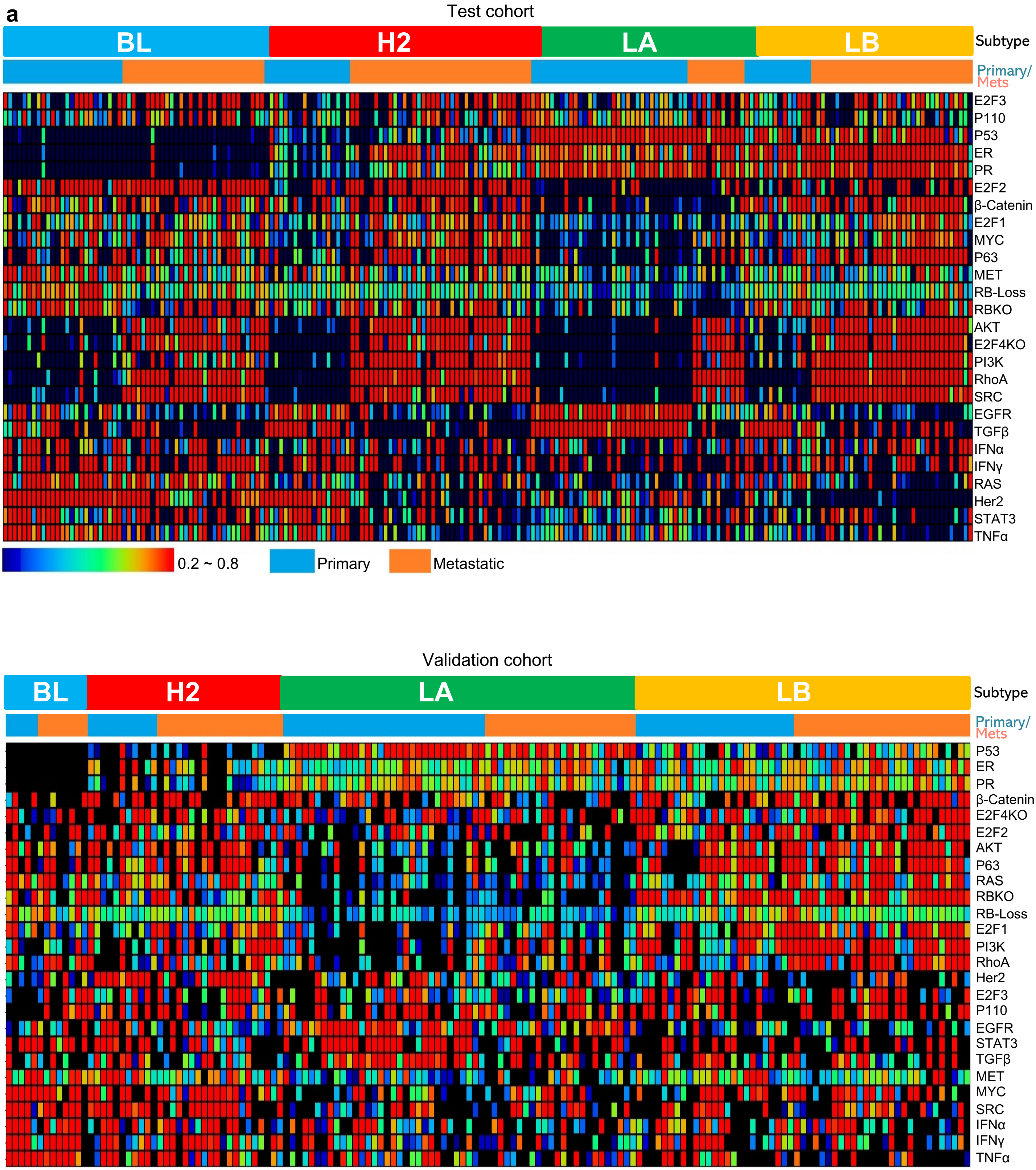

Supplemental Fig. S9a

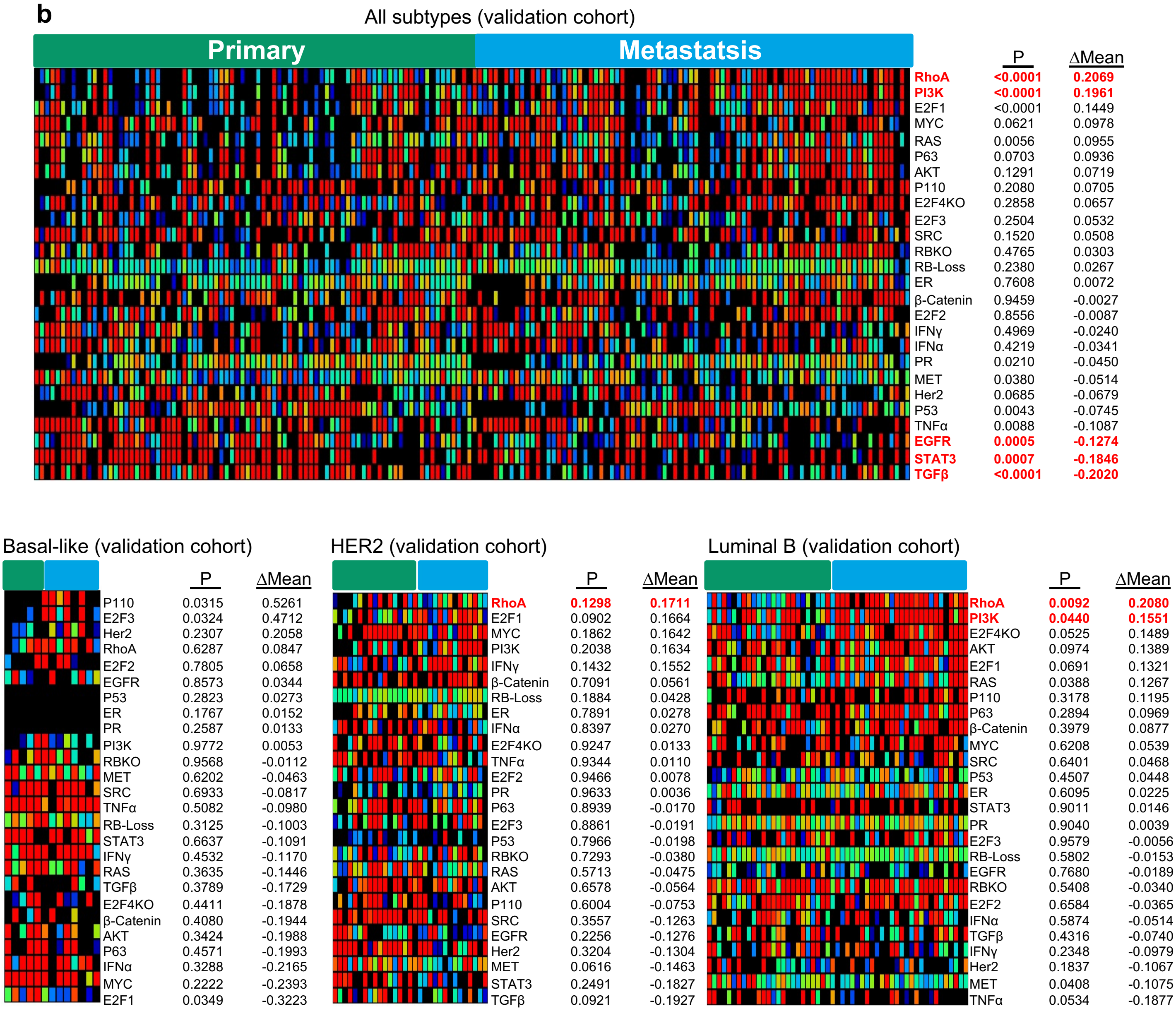

Supplemental Fig. S9b

c

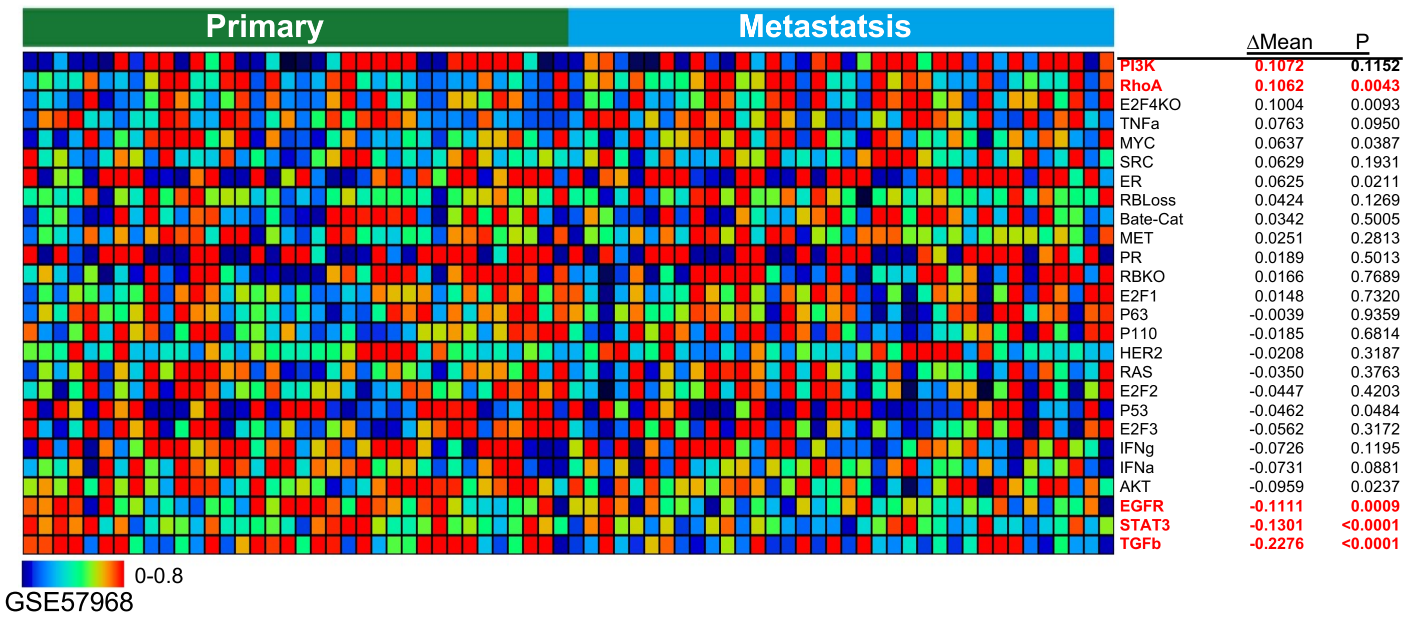

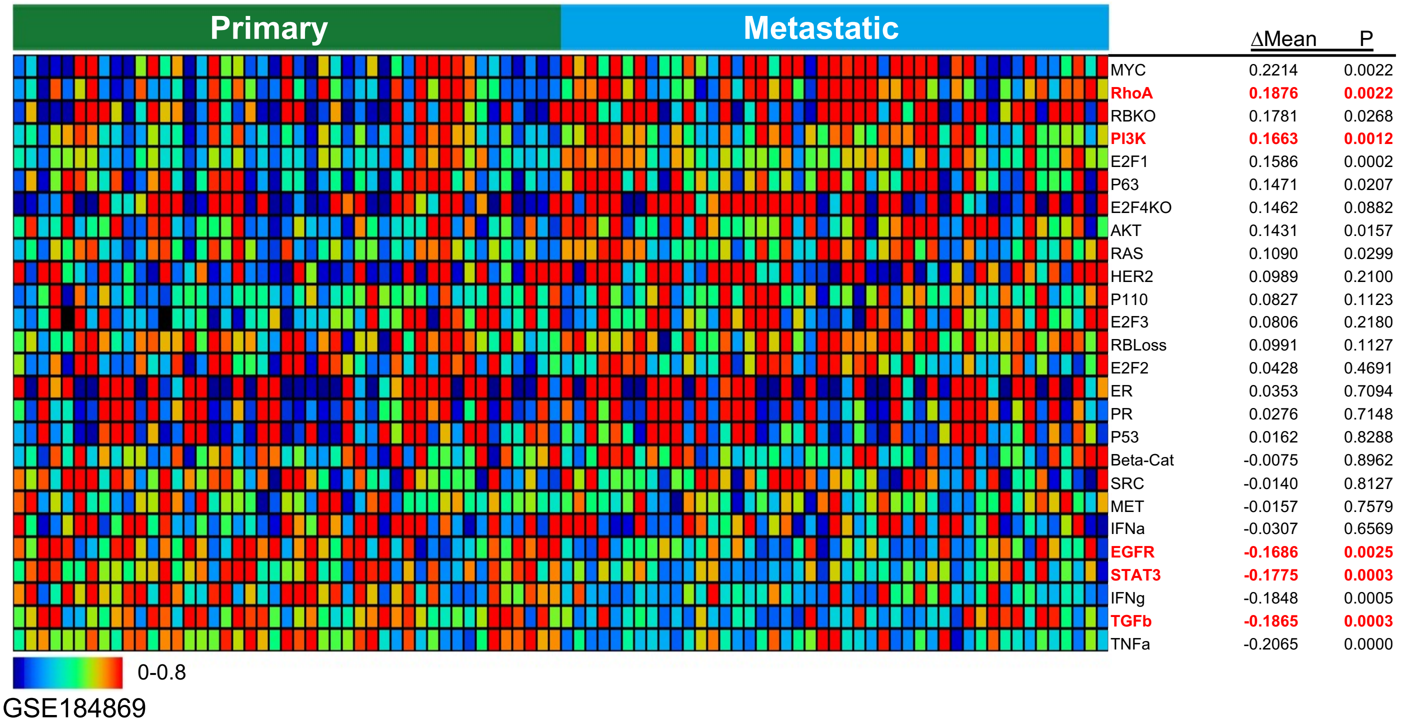

Supplementary Figure S9 Related to Figure 7. a. Activity of 26 signaling pathways and signatures in breast cancer samples segregated into molecular subtypes by PAM50 in primary vs metastatic tumors in test (top) and validation (bottom) cohorts. **b.** Activity of the 26 signaling pathways in all breast cancer samples or in each subtype in the validation cohort. Luminal A analysis of the validation cohort is shown in **Figure 7b**. **c.** Activity of the 26 signaling pathways in all breast cancer samples in two additional cohorts: GSE57968 with 36 and GSE184869 with 45 paired primary and metastatic tumors. Pathways denoted in red are consistently up- or down-regulated in metastasis in the test, validation and two additional cohorts.

Supplemental Fig. S9c

a RALBP1 ARHGAP4 RHOA PFN1 CDK5 VAV1 CHN1 PLD1 PIK3CA MYL2 RPS6KB1 CDC42 PIP5K1B ARHGAP5 ARHGEF5 PIK3CB ARHGEF1 GSN VCL LIMK1 OPHN1 MAP3K1 ARHGAP6 TRIO PIP5K1A ARFIP2 DIAPH1 TLN1 BAIAP2 PIK3R1 PPP1R12B WASF1 ARHGAP1 PAK1 RAC1 PIK3CG CDK5R1 PDGFRA ROCK1 NCF2 WASL ARHGEF11

b

RhoRacCDC42-pathway missense mutations in total primary vs metastatic BC from MSKCC

Available gene	2018-Primary Samples				2018-Metastasis Samples					2022-Metastasis Samples				
	Total Pri Sa No.	Pri Mt No.	Mis-sense-Mt	% mis-sense/ total	Total Met sa No.	Met Mt No.	Mis-sense-Mt	% mis-sense/ total	P value (vs.2018 pri)	Total Met Sa No	Met Mt No.	Mis-sense-Mt	% mis-sense/ total	P value (vs.2018 pri)
MAP3K1	902	124	22	2.44	978	96	26	2.66	0.7724	641	68	12	1.87	0.4872
PAK1	861	4	4	0.46	952	8	7	0.74	0.5533	628	0	0	0.00	0.1429
PDGFRA	862	6	5	0.58	952	19	17	1.79	0.0294	628	12	10	1.59	0.0662
PIK3CA	901	387	369	40.95	1013	414	400	39.49	0.5137	673	288	276	41.01	>0.9999
PIK3CG	861	11	11	1.28	952	14	13	1.37	>0.9999	628	10	10	1.59	0.6597
RAC1	861	3	3	0.35	952	2	2	0.21	0.6731	628	3	2	0.32	>0.9999
RHOA	861	4	4	0.46	952	14	13	1.37	0.0528	628	10	10	1.59	0.0308

c

PIK3CA hotspot Pmut analysis in total primary vs metastatic BC from MSKCC

8 Top-Mt-Position	Main protein variant (Hotspot)	2018 Mutation									2022 Mutation					
		Total-Mt-No.	Hot-spot No.	Mis-sense -Mt	Pri	TRUE	Pri-TURE %	Met	TRUE	Met-TURE %	Total-Mt-No.	Hot-Spot No.	Mis-sense _Mt	Met	TRUE	MET-TURE %
**345**	**N345K**	50	50	50	28	**28**	100.0	22	**22**	100.0	10	10	10	10	**10**	100.0
**420**	**C420R**	22	21	21	12	**11**	91.7	10	**10**	100.0	9	9	9	9	**9**	100.0
**453**	**E453K**	11	8	11	3	**0**	0.0	8	**0**	0.0	10	10	10	10	**0**	0.0
**542**	**E542K**	100	96	100	47	**46**	97.9	53	**52**	98.1	34	34	34	34	**34**	100.0
**545**	**E545K**	162	151	162	75	**4**	5.3	87	**5**	5.7	74	74	74	74	**0**	0.0
**546**	**Q546R**	18	7	18	10	**6**	60.0	8	**6**	75.0	4	3	4	4	**3**	75.0
**726**	**E726K**	22	22	22	7	**0**	0.0	15	**0**	0.0	10	10	10	10	**0**	0.0
**1047**	**H1047R**	290	249	288	143	**2**	1.4	147	**0**	0.0	87	82	87	87	**0**	0.0

Supplemental Fig. S10a-c

d

RhoRacCDC42-pathway's CNA in total BC from MSKCC datasets

PAK1, PIK3CA, CDC42, MAP3K1, PDGFRA, PIK3CB, PIK3CG, PIK3R1, RAC1, RHOA

Sample/Patient	2018 Primary	2018 Metastasis	2022 Metastasis
**Total Samples**	861	952	628
**PAK1-gain**	63 (7.3%)	104 (10.9%)	72 (11.5%)
**P (vs. 2018 Primary)**		0.0091	0.0078
**Total Patients**	837	869	534
**PAK1-gain**	63 (7.5%)	97 (11.2%)	63 (11.8%)
**P (vs. 2018 Primary)**		0.0101	0.0094

e

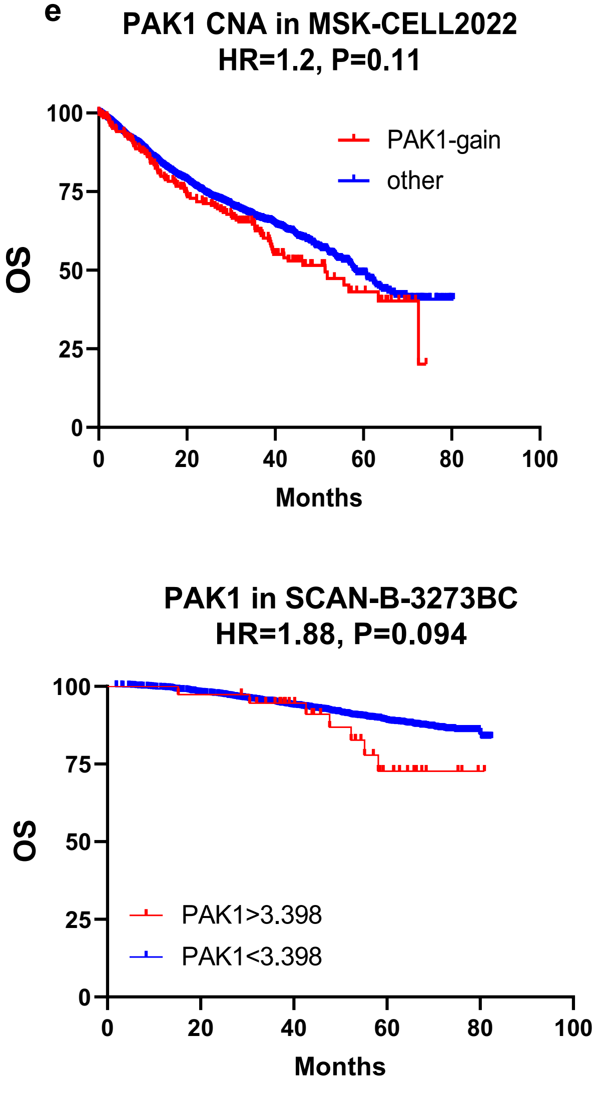

f

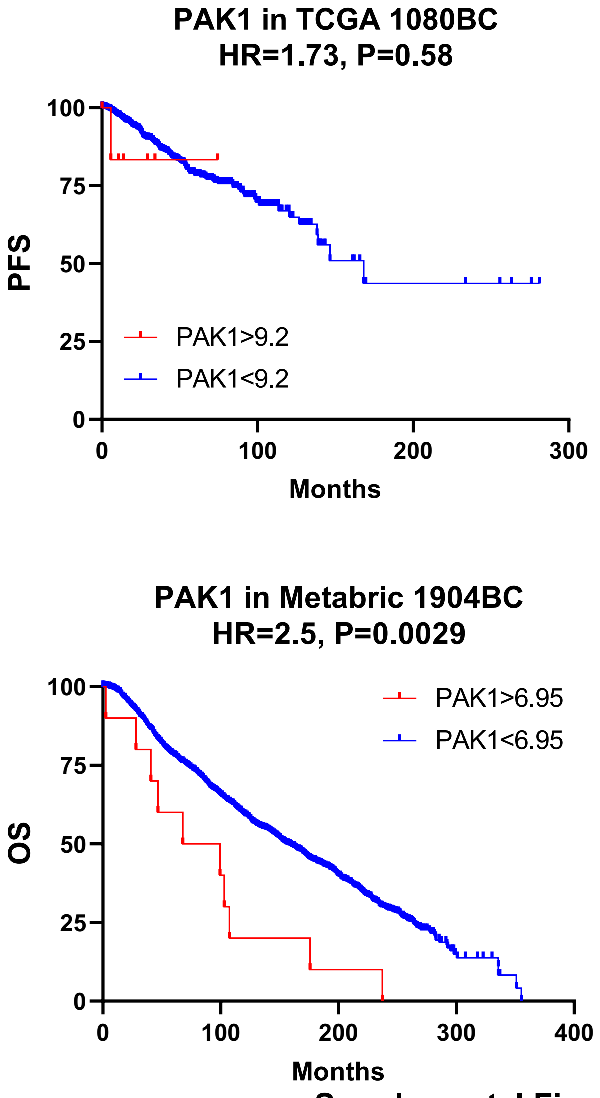

Supplemental Fig. S10d-f

Supplementary Figure S10 Related to Figure 7. Analysis of mutations and copy number alterations (CNAs) in 42 Rho/Rac1/CDC42 signaling genes in paired primary vs metastatic breast cancer samples in the MSKCC2018 and MSKCC2022 databases. **a.** List of the 42 Rho/Rac1/CDC42 genes analyzed herein. **b.** Of the 42 genes, mutation data on only 7 are available. Significant increases in missense mutations in primary vs metastases from MSKCC2018 and the metastases from MSKCC2022 datasets in PDGFRA and RHOA. Notably, no significant increase in mutation rate was observed following Pmut analysis, which predicts the likelihood of pathological mutations in missense mutations (TRUE or FALSE) but not in "Other" mutations, which include nonsense mutations, coding sequence variant frame shift, deletion in frame, insertion intron variant, splice acceptor variant, splice donor variant, stop gained, stop lost, etc. Note that the vast majority of mutations in MAP3K1 falls into the "Other" category and no significant enrichment in pathological mutation rate is seen in metastasis. **c.** Hot-spot mutational analysis of PIK3CA in metastasis vs primary tumors shows no increase in mutation rate in any of the indicated hot-spots. **d.** A total 474 genes were found in the CNA data of MSK2018 and 540 genes in MSK2022 including 10 on the Rho/Rac1/CDC42 gene list. Of these, only PAK1 showed significant increase in copy number gain in both metastatic patients and samples relative to primary tumors. **e.** PAC1 copy number gain does not correlate with significantly worse prognosis. **f.** High PAK1 mRNA expression correlates with worse prognosis in two different OS cohorts but not in the TCGA Progression free survival (PFS) dataset.

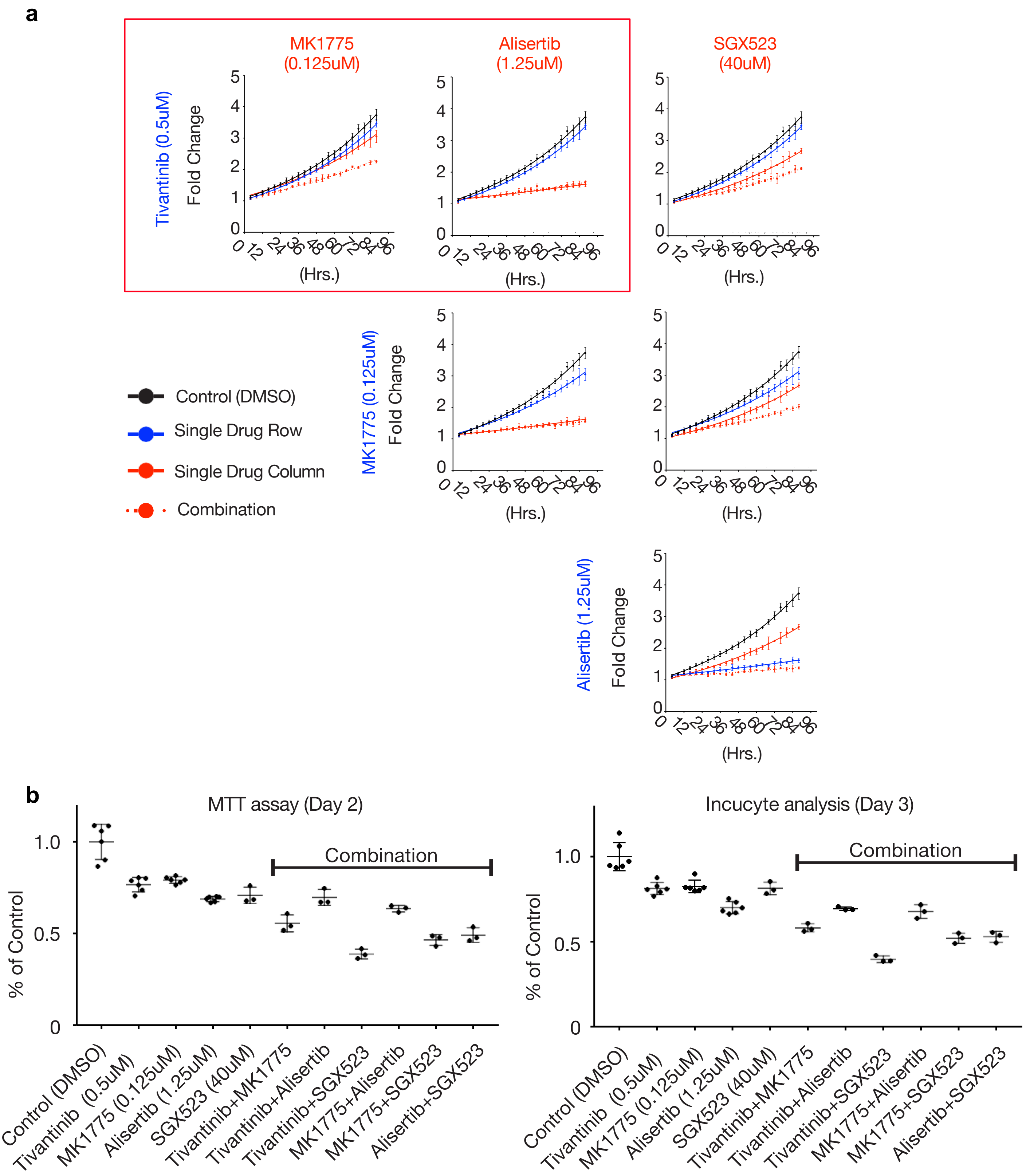

Supplemental Fig. S11a-b

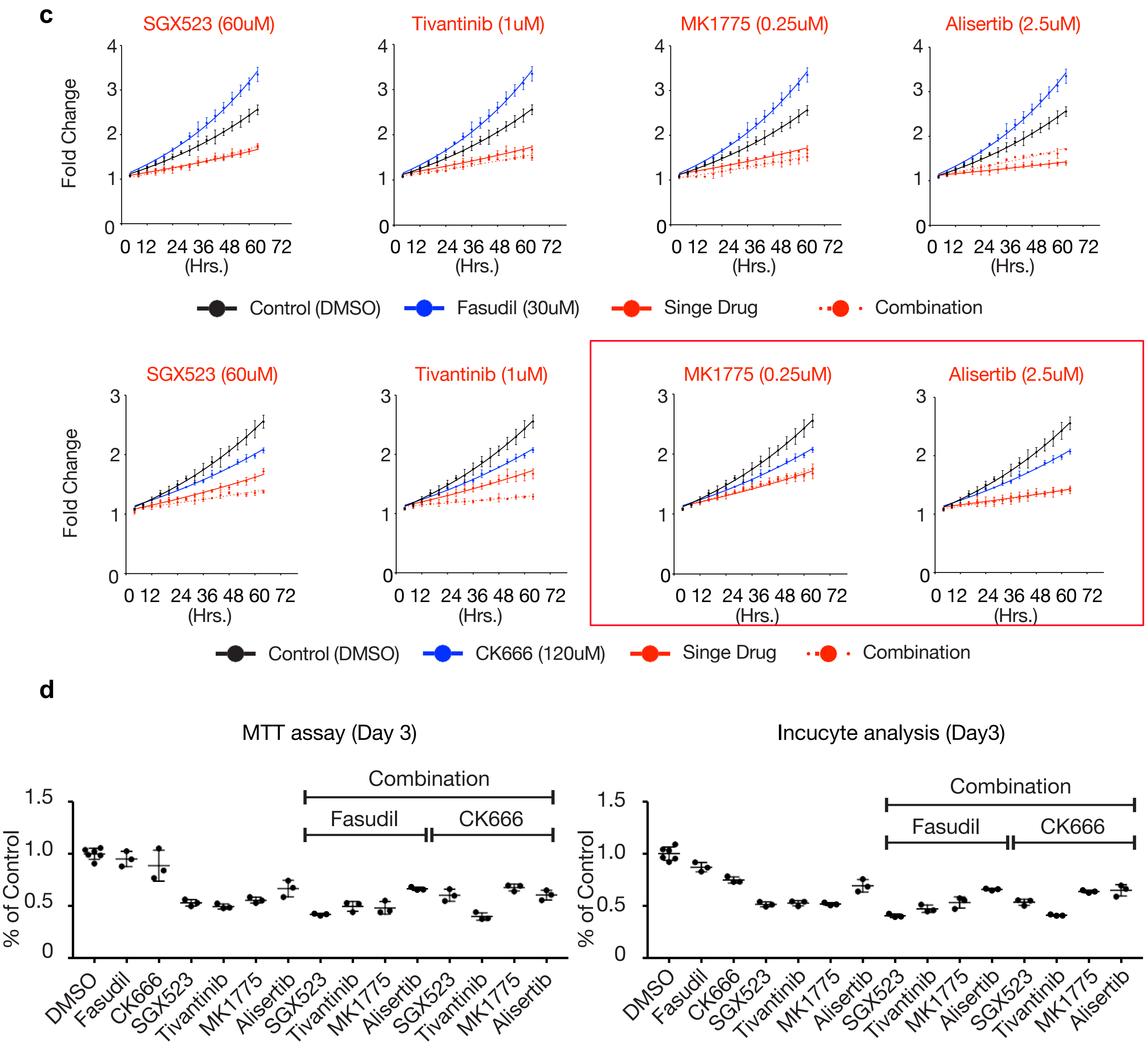

Supplemental Fig. S11c-d

e

INCUCYTE proliferation summary						
	Fausudil	CK666	MK1775	Alisertib	Tivantinib	SGX523
Fausudil			add/syn	antagonistic	add/syn	NC
CK666			NC	NC	add/syn	add/syn
MK1775	add/syn	NC		NC	add/syn	add/syn
Alisertib	antagonistic	NC	NC		NC	add/syn
Tivantinib	add/syn	add/syn	add/syn	NC		add/syn
SGX523	NC	add/syn	add/syn	add/syn	add/syn	
Notes:				add/syn=additive/synergistic		
				NC=no change		

migration inhibitors

f

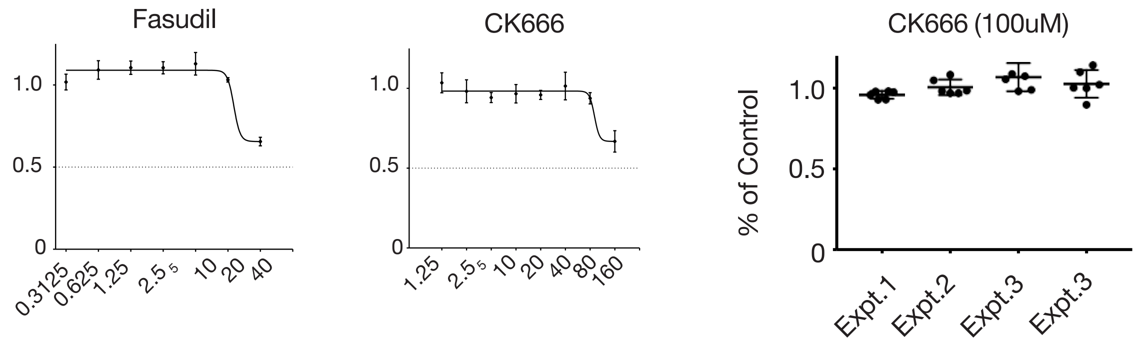

g

INCUCYTE migration assays

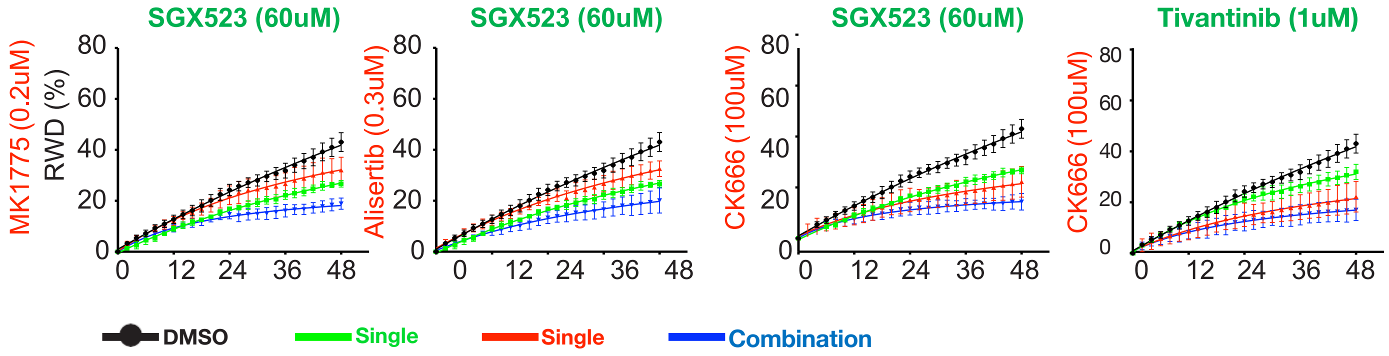

Supplementary Figure S11 Related to Figure 8b-c. Impact of inhibitors against two S-drivers vs S- plus M-specific drivers on cell proliferation and migration. **a.** Growth kinetics and viability of MDA-MB-436 cells treated with the indicated inhibitors that target RB-deficient cells and cMET using Incucyte analysis. **b.** Growth response and viability of MDA-MB-436 cells treated with indicated inhibitors against RB-deficient cells and cMET using MTT assays on day 2 on a replicate plate not used for Incucyte, or Incucyte analysis after 3 days. **c.** Growth response and viability of MDA-MB-436 cells treated with indicated S- and M-specific inhibitors alone or in combination for 3 days using Incucyte analysis. Red box demarcates combination treatments with CK666 plus MK1775 versus CK666 plus Alisertib. **d.** Summary of drug effects using MTT assays or Incucyte. **e.** Summary of additive effects/synergism or antagonism between the indicated drugs using Incucyte analysis based on panels c-d. **f.** Titration of the two migration inhibitors Fasudil and CK666. For the combination analysis on cell migration, CK666 concentration of 100uM, in which no effect on cell proliferation is evident, was used. **g.** Impact of indicated single and combination drug analysis on Incucyte-based migration analysis. Error bars in all panels represent SD.

Supplemental Fig. S11e-g

Uncropped images of western blots for supplemental figures

Fig. S6

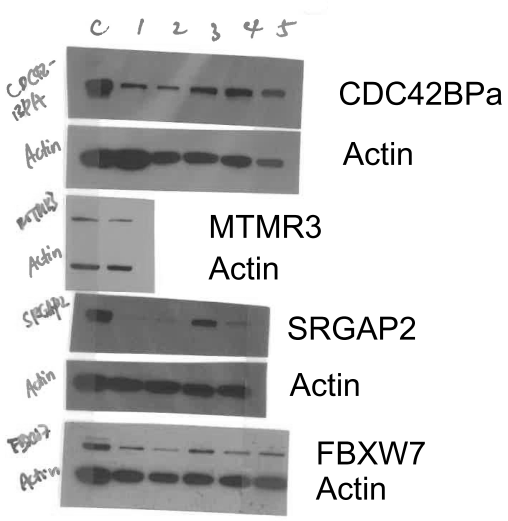

Fig. S7d

MDA-MB-468

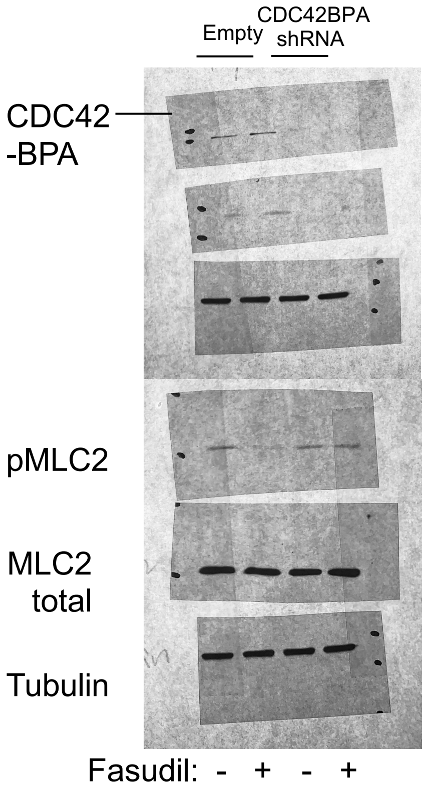

Fig. S8a

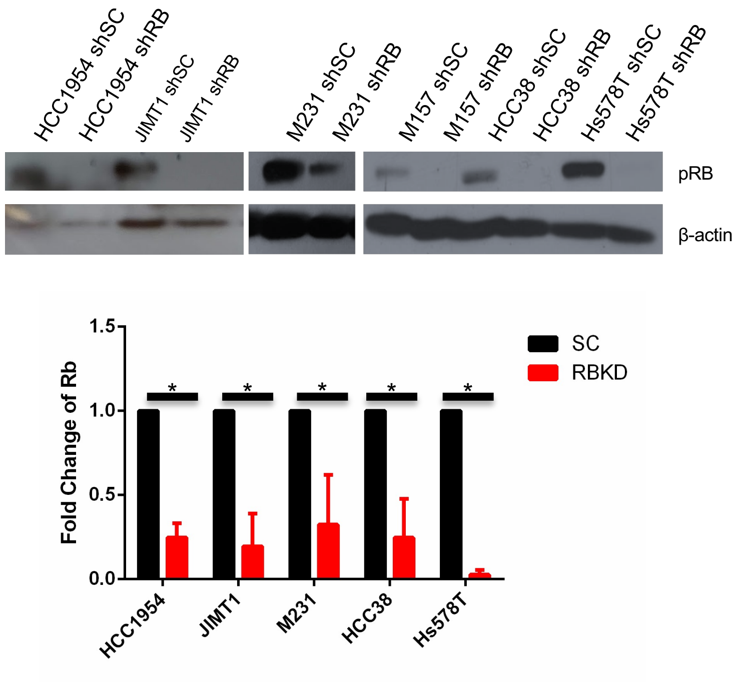

Fig. S8b

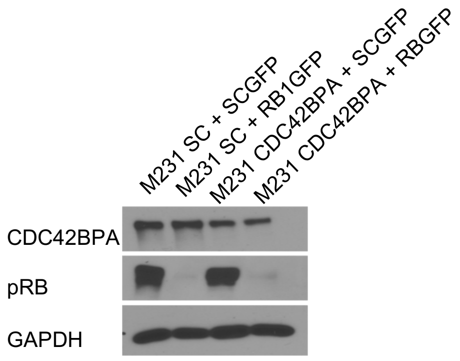

Supplement: Supplementary file 1 — Supplementary Information [file 41467_2023_39935_MOESM1_ESM.pdf]
