# Supplementary material for: Distinct shared and compartment-enriched oncogenic networks drive primary versus metastatic breast cancer
Source: Nat Commun. 2023 Jul 18;14:4313. doi: 10.1038/s41467-023-39935-y (PMC10354065; doi:10.1038/s41467-023-39935-y)
Supplement: Supplementary file 3 — Description of Additional Supplementary Files [file 41467_2023_39935_MOESM3_ESM.pdf]

Description of Additional Supplementary Files

Supplementary Data 1 : related to Fig. 1. Gene-centric common insertion sites (gCIS; top row; black boxes) identified at highly stringent filtered_clonal level in biopsies from primary mammary tumors (column A) following sleeping beauty mutagenesis screens on Rb-deletion background in MMTV-Cre:Rb$^{f/f}$:T2/Onc3a:R26^{lsl}_^{SB11} and MMTV-Cre:Rb$^{f/f}$:T2/Onc3b:R26^{lsl}_^{SB11} female mice.

Supplementary Data 2 : related to Fig. 1. gCIS identified at highly stringent filtered_clonal level from whole lung metastases (column A) following sleeping beauty mutagenesis screens on Rb-deletion background in MMTV-Cre:Rb$^{f/f}$:T2/Onc3a:R26^{lsl}_^{SB11} and MMTV-Cre:Rb$^{f/f}$:T2/Onc3b: R26lsl_SB11 female mice.

Supplementary Data 3 : related to Fig. 2. gCIS identified at medium stringent filtered_subclonal level in biopsies from primary mammary tumors (column A) following sleeping beauty mutagenesis screens on Rb-deletion background in MMTV-Cre:Rb$^{f/f}$:T2/Onc3a:R26^{lsl}_^{SB11} and MMTV-Cre:Rbf/f:T2/Onc3b: $^{R26lsl\_SB11}$ female mice.

Supplementary Data 4 : related to Fig. 2. gCIS identified at medium stringent filtered_subclonal level from whole lung metastases (column A) following sleeping beauty mutagenesis screens on Rb-deletion background in MMTV-Cre:Rb$^{f/f}$:T2/Onc3a:R26^{lsl}_^{SB11} and MMTV-Cre:Rbf/f:T2/Onc3b: $^{R26lsl\_SB11}$ female mice.

Supplementary Data 5 : related to Fig. 2. Integration site analysis of sleeping beauty transposons demonstrating clonal relationship between mammary and lung lesions from the same mice. Shown are the nucleotide sequence at the integration sites in primary and lung tumors for the indicated genes from the filtered_subclonal gCIS analysis. For example, for cMet primary filtered_subclonal pair 1, there are transposons in 4 different integration sites in primary tumors, one of which gave rise to 9 different lung metastases with identical integration sites between nucleotides 17436202 and 17436203 in intron 1.
