# Supplementary material for: Distinct shared and compartment-enriched oncogenic networks drive primary versus metastatic breast cancer
Source: Nat Commun. 2023 Jul 18;14:4313. doi: 10.1038/s41467-023-39935-y (PMC10354065; doi:10.1038/s41467-023-39935-y)
Supplement: Supplementary file 5 — Reporting Summary [file 41467_2023_39935_MOESM5_ESM.pdf]

nature portfolio

Corresponding author(s): Eldad Zacksenhaus

Last updated by author(s): 25/4/23

Reporting Summary

Nature Portfolio wishes to improve the reproducibility of the work that we publish. This form provides structure for consistency and transparency in reporting. For further information on Nature Portfolio policies, see our Editorial Policies and the Editorial Policy Checklist.

Statistics

For all statistical analyses, confirm that the following items are present in the figure legend, table legend, main text, or Methods section.

n/a	Confirmed	
☐	☒	The exact sample size (*n*) for each experimental group/condition, given as a discrete number and unit of measurement
☐	☒	A statement on whether measurements were taken from distinct samples or whether the same sample was measured repeatedly
☐	☒	The statistical test(s) used AND whether they are one- or two-sided<br>*Only common tests should be described solely by name; describe more complex techniques in the Methods section.*
☒	☐	A description of all covariates tested
☒	☐	A description of any assumptions or corrections, such as tests of normality and adjustment for multiple comparisons
☐	☒	A full description of the statistical parameters including central tendency (e.g. means) or other basic estimates (e.g. regression coefficient) AND variation (e.g. standard deviation) or associated estimates of uncertainty (e.g. confidence intervals)
☐	☒	For null hypothesis testing, the test statistic (e.g. *F*, *t*, *r*) with confidence intervals, effect sizes, degrees of freedom and *P* value noted<br>*Give P values as exact values whenever suitable.*
☒	☐	For Bayesian analysis, information on the choice of priors and Markov chain Monte Carlo settings
☒	☐	For hierarchical and complex designs, identification of the appropriate level for tests and full reporting of outcomes
☒	☐	Estimates of effect sizes (e.g. Cohen's *d*, Pearson's *r*), indicating how they were calculated

Our web collection on statistics for biologists contains articles on many of the points above.

Software and code

Policy information about availability of computer code

Data collection	Image J was used for data collection/measurments of lung metastases
Data analysis	Microsoft Excel Office 16.54/GraphPad Prism 9/Image J 1.53awere used to analyze results and generate jitter plots<br>Publically available datasets used were indicated by citation (reference) and GSE number.

For manuscripts utilizing custom algorithms or software that are central to the research but not yet described in published literature, software must be made available to editors and reviewers. We strongly encourage code deposition in a community repository (e.g. GitHub). See the Nature Portfolio guidelines for submitting code & software for further information.

Data

Policy information about availability of data

All manuscripts must include a data availability statement. This statement should provide the following information, where applicable:

- Accession codes, unique identifiers, or web links for publicly available datasets
- A description of any restrictions on data availability
- For clinical datasets or third party data, please ensure that the statement adheres to our policy

Bioinformatice analyses were performed on publicly available data set. GSE accession numbers and hyperlinks are provided. Sequence data from the SB insertional mutagenesis screens described in this study were deposited in the Gene Expression Omnibus (GEO) database under accession code GSE232167 (see DATA AVAILABILITY statement).

Human research participants

Policy information about studies involving human research participants and Sex and Gender in Research.

Reporting on sex and gender	n/a
Population characteristics	n/a
Recruitment	n/a
Ethics oversight	n/a

Note that full information on the approval of the study protocol must also be provided in the manuscript.

Field-specific reporting

Please select the one below that is the best fit for your research. If you are not sure, read the appropriate sections before making your selection.

☒ Life sciences ☐ Behavioural & social sciences ☐ Ecological, evolutionary & environmental sciences

For a reference copy of the document with all sections, see nature.com/documents/nr-reporting-summary-flat.pdf

Life sciences study design

All studies must disclose on these points even when the disclosure is negative.

Sample size	No sample-size calculation was performed. In general, experiments were performed in 3 or more biological replicates, each in triplicates or more, and statistical significance was calculated as indicated, with P<0.05 considered significant. SB screens and genetic analysis were performed on large number of mice to increase the power of analysis and the frequency of representative insertions. For transplantation experiments, we used 6 or more mice per group to obtain statistically significant results.
Data exclusions	No data were excluded from the analyses
Replication	Experiments were performed 3 or more times when possible as indicated to verify the reproducibility of the experimental findings. There are no findings that were not replicated.
Randomization	For gCIS analysis, biopsies were taken randomly from large tumors; metastases were collected whole and each analyzed separately.
Blinding	gGIS, GSEA and other bioinformatics analysis were performed on samples without prior knowledge of expected results. Tumor cell injection and monitoring were performed by different researchers. NSG mice were randomly selected for injections of experimental or control cells. Blinding for other experiments was not possible as the same person performed and analyzed experiments

Reporting for specific materials, systems and methods

We require information from authors about some types of materials, experimental systems and methods used in many studies. Here, indicate whether each material, system or method listed is relevant to your study. If you are not sure if a list item applies to your research, read the appropriate section before selecting a response.

Materials & experimental systems

n/a	Involved in the study	
☐	☒	Antibodies
☐	☒	Eukaryotic cell lines
☒	☐	Palaeontology and archaeology
☐	☒	Animals and other organisms
☐	☒	Clinical data
☒	☐	Dual use research of concern

Methods

n/a	Involved in the study	
☒	☐	ChIP-seq
☒	☐	Flow cytometry
☒	☐	MRI-based neuroimaging

Antibodies

Antibodies used	Primary antibodies: Rabbit anti-human RB1 (Cell Signalling Technologies, cat. 9313); rabbit anti-FBXW7 (EAP3553, Elabscience), rabbit anti-SRGAP2 (GTX130797, GeneTex), mouse anti-CDC42BPA (MRCKα) (sc-374568, Santa Cruz Biotechnology), mouse anti-MTMR3

March 2021

	(sc-393779, Santa Cruz Biotechnology), anti-pMLC2-Thr18/Ser19 (#3674), total MLC2 (#3672; Cell Signaling Technology), MRPL37 (ABcam 224467), GAPDH (sc-47724), rabbit anti-Tubulin (#2148, Cell Signaling Technology), and mouse anti-Actin (JLA20, Developmental Studies Hybridoma Bank). Secondary antibodies: anti-rabbit IgG-HRP (Cell Signalling Technologies, cat. 7074), anti-mouse IgG-HRP (Cell Signalling Technologies, cat. 7076).
Validation	For antibodies listed above, validations and citations can be found on the manufacturer's website. In addition, our shRNA data shows specific depletion of the targets, thus also validating specificity of these antibody.

Eukaryotic cell lines

Policy information about cell lines and Sex and Gender in Research

Cell line source(s)	Human breast cancer cell lines: MDA-MB-231, MDA-MB-436, MDA-MB-468, HCC38, Hs57T and MCF7 were maintained in DMEM containing 10% FBS and 1% PEST, at 37oC with 5 % CO2. MDA-MB-436, and MDA-MB-231 were kindly obtained from the late Dr. Mona Gauthier, and the remaining were purchased as previously described37,87, from the American Type Culture Collection (ATCC). Human embryonic kidney cells, HEK293T, obtained from Dr. Jason Moffat129, were cultured as above.
Authentication	None of the cell lines were authenticated.
Mycoplasma contamination	PCR based analysis confirmed the breast cancer lines used in this study were mycoplasma-free.
Commonly misidentified lines (See ICLAC register)	Did not use any commonly misidentified lines

Animals and other research organisms

Policy information about studies involving animals; ARRIVE guidelines recommended for reporting animal research, and Sex and Gender in Research

Laboratory animals	Composite mice were on mixed background,; bred in house and were reported previously by the authors. Fbxw7f/f mice were obtained from JAX; NSG female mice from UHN institute's mouse colony. Age of mice: for SB screens, mice were monitored till they developed tumors (6-12 months); for transplantation experiments – NSG mice were injected with modified MDA-MB-436 TNBC cells at 6-8 weeks of age, and monitored for tumor development for 1-2 months till primary tumors reached 2 cm in diameter. Mice were maintained on standard chow (Harlan-TEKLAD LM-4857912.15) ad libitum, at 22-24°C, 36-40% humidity, and 12 hr dark/light cycle.
Wild animals	This study did not involve wild animals.
Reporting on sex	Finding are directly relevant to breast cancer - but the principle of P, S and M-drivers may apply to other types of cancer such as colon cancer in both sexes.
Field-collected samples	This study did not involve samples collected from the field.
Ethics oversight	Research Institute Animal Research Committee at University Health Network, Canada.

Note that full information on the approval of the study protocol must also be provided in the manuscript.

Clinical data

Policy information about clinical studies

All manuscripts should comply with the ICMJE guidelines for publication of clinical research and a completed CONSORT checklist must be included with all submissions.

Clinical trial registration	n/a. Note that we used publicly available data on breast cancer patients but the study did not involve new patient sample collection/ treatment.
Study protocol	n/a
Data collection	n/a
Outcomes	n/a
